# Mobile Heart Rate Variability Biofeedback as a Complementary Intervention After Myocardial Infarction: a Randomized Controlled Study

**DOI:** 10.1007/s12529-021-10000-6

**Published:** 2021-05-18

**Authors:** Anja Limmer, Martin Laser, Astrid Schütz

**Affiliations:** 1 Department of Psychology, University of Bamberg, Bamberg, Germany; 2 Praxis Dr. med. Martin Laser, Nuremberg, Germany

**Keywords:** Myocardial infarction, Heart rate variability biofeedback, Secondary prevention, Risk factors, Self-efficacy

## Abstract

**Background:**

To enhance effective prevention programs after myocardial infarction (MI), the study examined the effects and feasibility of mobile biofeedback training on heart rate variability (HRV-BF).

**Methods:**

Forty-six outpatients aged 41 to 79 years with a documented MI were randomized to HRV-BF versus usual care. Generalized estimating equation (GEE) analyses were performed to test improvements in measures of short- and long-time HRV, namely, the standard deviation of the normal-to-normal intervals (SDNN) and well-being after 12 weeks of HRV-BF.

**Results:**

There were intervention effects for short-time HRV (*d* > 0.4, *p* < 0.04), which were partly replicated in the GEE models that accounted for control variables: In the HRV-BF group, the high-frequency HRV (group × time interaction: *β* = 0.59, *p* = 0.04) compensated for significantly lower baseline levels than the group with usual care. In an optimal dose sample (on average two HRV-BF sessions a day), SDNN significantly increased after HRV-BF (*p* = 0.002) but not in the waitlist control group. Compensatory trends of HRV-BF were also found for high-frequency HRV and self-efficacy. No adverse effects of the intervention were found but neither were effects on long-time HRV measures.

**Conclusion:**

The results showed the feasibility of self-guided HRV-BF for almost all post-MI patients. HRV-BF as an adjunctive behavioral treatment increased HRV, which is an indicator of lower cardiovascular risk, and self-efficacy, which suggests heightened psychological resilience. These benefits warrant confirmation and tests of sustainability in larger studies.

**Trial Registration:**

The trial has not been registered due to its starting point in 2017 predating the publication of the applicable CONSORT extension for reporting social and psychological intervention trials in 2018.

**Supplementary Information:**

The online version contains supplementary material available at 10.1007/s12529-021-10000-6.

## Introduction

Diseases of the circulatory system, most notably coronary heart disease, remain the major cause of death in Europe and beyond [1]. Despite a slight decrease in mortality rates in most industrialized countries [2], the potential for coronary disease prevention has not been exhausted [3], which holds true in particular for secondary prevention [4].

On the one hand, there is broad consensus that prevention is crucial for short- and long-term outcomes in coronary artery disease and the guidelines recommend a multidimensional approach that includes multimodal behavioral interventions (class I, level A), including relaxation training [5, 6]. On the other hand, there are still problems in implementing prevention measures over and above standard medication [7]. Challenges include the long-term maintenance of lifestyle changes that require extra time and skills [6]. Furthermore, the low attendance rates that are common at preventive programs may be due to the reluctance of both patients and professionals to address non-physiological aspects of health, despite the evidence that intensifying efforts in improving secondary prevention measures would contribute to a reduction in the mortality burden [2].

**Heart Rate Variability and Cardiovascular Health.** Heart rate variability (HRV) describes “the complex modification of the heart rate by the coordination of autonomic, respiratory, circulatory, endocrine and mechanical influences over time.” [8] (p. 1) These fluctuations in heart rate can be measured by change in the time intervals between consecutive heartbeats [9]. A variety of studies have shown that both mental and somatic problems (e.g., asthma, diabetes, cardiovascular disease, and depression) are related to low HRV [10]. The importance of HRV for cardiovascular health was made particularly evident in Kleiger et al.’s [11] study, which showed that a quantification of HRV (i.e., the standard deviation of the RR intervals in sinus rhythm using 24 h recordings) was an independent predictor of mortality after acute MI.

Numerous studies have since supported the notion that the analysis of HRV can make a significant contribution to cardiac risk stratification [12]. The association between reduced heart rate variability and cardiac mortality is also reflected in short-term measurements [13–15]. Recently, HRV has been more widely adopted to describe the autonomic control of the heart rate in the short term [8] and as a surrogate index for the effects of secondary prevention strategies [12].

Besides the well-established role of decreased HRV as a predictor of mortality over and above other cardiovascular risk factors, decreased HRV has also been discussed to precede these factors: Reviewing the literature, Wulsin et al. [15] found evidence that biological factors, such as genetic vulnerability, health behaviors (e.g., smoking), and chronic stress [16], are related to reduced HRV. Both perspectives on decreased HRV, either as an independent risk marker or as the final common pathway linking major risk factors to cardiovascular disease or death, lead to the same conclusion, namely, that improving HRV may serve to prevent or at least minimize cardiovascular risk.

**HRV Biofeedback Training.** Considering the relationship between HRV and many core clinical features of health mentioned above, treatments aimed at increasing HRV have gained attention [9]. As one behavioral intervention, biofeedback training helps individuals alter physiological processes (e.g., heart rate). Besides learning processes, cognitive-attributional changes such as the improvement of self-efficacy, defined as people’s beliefs in their capabilities [17], are suggested to be a key therapeutic mechanisms in biofeedback [18]. Biofeedback training on HRV combines slow breathing and feedback on the resulting changes in HRV. The desired effect of rhythmical heart rate oscillations with a multiplied amplitude can be attributed to the confluence of several physiological mechanisms [19]. Besides the strengthening of the baroreflex and resonance in the relationship between heart rate, blood pressure, and breathing rate (restored autonomic homeostasis), central effects in the frontal cortex and cholinergic anti-inflammatory processes have been most frequently discussed to account for the benefits of HRV-BF [9, 10]. In the context of MI, especially the modulation of inflammatory responses could have a beneficial effect, given the increasing evidence linking inflammation to the development and manifestation of atherosclerosis. [20].

HRV-BF has consistently been shown to increase HRV measures and aspects of psychological functioning, such as cognitive functioning or emotional regulation (e.g., anxiety and depression), in the treatment of cardiovascular diseases [19, 21, 22]. Yu et al. [23] confirmed the sustainability of these effects of HRV-BF and, moreover, its long-term effect on the cardiovascular prognosis of patients with coronary artery disease in line with Cowan et al. [24]. However, the HRV-BF interventions that have been studied have varied in terms of additional (e.g., psychoeducational) contents and the number and duration of sessions. Thus, additional research is needed to identify the effects for specific clusters of disorders [19]. Further, research has mainly been based on stationary biofeedback equipment, inseparably linked with a limited number of prescheduled sessions in the presence of HRV-BF experts, whereas technological advances have meanwhile increased the availability and flexibility of mobile or wearable HRV-BF devices [25]. To the best of our knowledge, the specific effects of mobile HRV-BF have not been examined in patients after acute MI.

In summary, HRV can be seen as a risk marker that is linked to a worse prognosis after MI on the one hand. On the other hand, HRV-BF promotes physiological and psychological well-being in patients with cardiovascular disorders. Nevertheless, there is a need for evidence on whether HRV-BF can improve HRV and other aspects of health in patients after MI in an outpatient treatment. The present study is aimed at examining the effects and feasibility of mobile HRV-BF as a supplement to secondary preventive interventions after MI. We hypothesized that HRV-BF as a complement to standard care in post-MI patients would improve aspects of psychological well-being (e.g., stress and self-efficacy) as well as HRV parameters that reflect cardiac autonomic balance or risk.

## Methods

### Study Population and Design

A randomized controlled design was adopted in a cardiological outpatient practice in Germany. Adult patients were assigned to either the HRV-BF group or the waitlist control group. Additional participants were recruited via newspaper articles and information sessions on cardiovascular disease prevention between 2017 and 2019. The prerequisite for participation was a previous MI (ascertained by the evaluation of cardiac troponin) that was documented in the patients’ records. Patients with known atrial fibrillation, continuous pacemaker stimulation, or psychopharmaceutical medication were excluded. A randomization sequence for the two groups was generated by a computer tool (sealed envelope; [26]) using randomly varying block sizes of 4, 6, and 8. The study was approved by a university institutional review board on 17 July 2017, and all participants provided written informed consent before enrolling in the study. A case number calculation was conducted using G*Power 3.1.9.2 software [27]). Assuming a medium effect size based on previous studies [28], at least 54 participants were required to achieve a power of 0.80 at *α* = 0.05 for changes in the primary outcome measure (SDNN). At admission, we documented sociodemographic data (e.g., age, gender) and clinical parameters (e.g., time since last MI, known comorbidities, and the intake of psychotropic drugs). Before and at the end of the HRV-BF or waiting interval of about 12 weeks, physiological and psychological measures were assessed.

### Intervention/HRV-BF Protocol

BF was practiced using a battery-powered handheld HRV-biofeedback device, the Qiu (BioSign, Germany), which measures the pulse via an optical sensor and calculates the HRV. The upper half of the spherical device provides continuous visual feedback via a stepless spectrum of colored light ranging from red (low HRV) to green (hight HRV). The device can be set at different levels of difficulty. Additionally, moving blue LED lights can be used to guide the breathing frequency at an individually adjustable pace. The device records the time of every training sequence and the complete heart rate curve. Data were read via a USB port and transmitted via email or at patient visits. In the 30 min introductory session, general information about HRV, relaxation, and the handling of the Qiu was provided, and participants practiced abdominal breathing. The HRV-BF sessions were guided by a certified expert in HRV-BF in quiet rooms in a cardiologist’s office. The participants were asked to practice at home, three 5 min sessions per day. The instructor recommended an even distribution of training over the day but highlighted that the training could be anytime or anywhere (e.g., at home, on a work break, on a train). Participants received handouts with instructions for the HRV-BF exercises and the handling of the device. They were instructed to breathe at their own resonance frequency (approximately 0.1 Hz [19]), guided by the correspondingly adjusted pacer of the Qiu, and to focus their attention on their breathing. Follow-up sessions with the HRV expert were arranged depending on the training progress after around 1, 3, and 6 weeks. After 12 weeks, there was a final session to evaluate the training results and discuss further steps of behavioral measures for promoting health. During the first 3 weeks of training, participants received a weekly phone call to check on their condition and possible problems.

The waitlist control group received standard medical care and had the opportunity to participate in the same HRV-BF program after the post-intervention assessment. All participants completed psychological questionnaires and 5 min HRV measurements as well as a 24 h Holter electrocardiogram (ECG) pre- and post-intervention. Medical records confirming MI were obtained with the participants’ permission.

### Data Collection and Outcome Measures

Twenty-four-hour Holter ECG recordings were obtained using a validated three-channel device (Lifecard CF, Spacelabs Healthcare, US). All Holter ECG studies were post-processed by a trained study collaborator using dedicated software (Pathfinder SL, Spacelabs Healthcare, UK). As the primary endpoint, we used the standard deviation of all normal RR intervals (long-time SDNN) in ms, which was automatically calculated for the recording period, as well as the mean systolic blood pressure (in mmHg).

The standardized short-term measuring protocol used the HRV-Scanner® software (version V3.07, Biosign, Germany) to analyze ECG raw signals from clamp electrodes on the wrists for 5 min at rest with a sampling rate of 500 Hz. An experienced user visually screened the HR data and corrected artifacts before transforming them into HRV indices. For the best comparability [14], we used SDNN (short-time SDNN) as a time-domain parameter as well as the frequency domain parameters high-frequency (HF, 0.15–0.4 Hz) and low-frequency (LF, 0.04 to 0.15 Hz) power in units of ms^2^ for the present analysis. These frequency domain indices have also been shown to be important outcomes for HRV-BF in the context of coronary artery disease [23]. As a further predictor of cardiovascular disorders [29], mean heart rate (per minute) was also assessed during the short-term measuring protocol along with breathing frequency (per minute).

During the baseline visit, we documented sociodemographic data (age, gender) and clinical parameters (e.g., time of the last MI and the intake of cardiovascular or psychotropic drugs). In addition, a screening score for emotional distress was assessed by two standardized items analogous to current guidelines [5]. For further secondary outcomes, participants completed a psychological screening with standardized self-report questionnaires before and after the intervention or waiting period. Overall psychological well-being was assessed with a German version of the World Health Organization Well-Being Index (WHO-5), which has previously shown adequate validity in assessing subjective well-being over time and between groups [30]. The WHO-5 consists of five positively phrased items for measuring subjective well-being during the last 2 weeks, rated on a 6-point Likert scale ranging from 5 (all of the time) to 0 (none of the time). The raw score therefore ranges from 0 to 25 where high scores signify better well-being, and low scores indicate mental health problems [30]. Self-efficacy was measured with the German version of the short scale for measuring general self-efficacy beliefs (ASKU) [31]. The scale comprises three items (e. g. “I am able to solve most problems on my own.”), which are rated on a 5-point Likert scale and summed up to a total score. The short scale has been shown to be reliable and valid for assessing individuals’ global confidence in dealing with demanding situations [31]. The validated Screening Scale of the Trier Inventory for Chronic Stress (SSCR-TICS) [32] was used to evaluate perceived stress in everyday life during the previous 3 months. The screening scale consists of 12 items (e.g., “I feel overwhelmed by my tasks”) that are scored on a five-point Likert scale ranging from 0 (labeled as “never”) to 4 (labeled as “always”). The resulting total score ranges from 0 to 48 points. Higher scores mean that stressors are experienced more often.

### Statistical Analysis

Baseline characteristics were compared across groups using Pearson’s Chi^2^ test, Fisher’s exact test, Student’s t test, or the Wilcoxon rank sum test, as appropriate. This was also done to compare participants who dropped out with those who remained. Besides skewness and kurtosis, the Shapiro–Wilk *W* test was used to evaluate the distribution of continuous variables. HRV indices, mean systolic blood pressure, and heart rate were log-transformed, and the Well-being Index was squared to improve the normality of the distributions.

Due to the relatively small sample size, univariate analyses were applied to get an overview of the effect sizes (Cohen’s *d*). To adjust for the dependency of the repeated measures within one participant, separate multivariate GEE analyses with time and group as categorial predictors were performed for each dependent variable. GEE is capable of handling missing data, which renders the method especially efficient for small samples [33]. Confounds were selected a priori and included age (years), gender (male/female), emotional distress, and the time lag since the last MI (years). In order to control for baseline differences, a term representing the interaction between group and measurement time was added to the models. When significant differences between groups were found, post hoc Bonferroni-adjusted comparisons of group and time differences were performed separately. In addition, post hoc GEE analyses were carried out for the “optimal dose” (i.e., for participants with at least 67% of the prescribed self-guided sessions of HRV-BF). All analyses were computed using STATA/SE 14.2.

## Results

As shown in the Electronic Supplementary Figure S1, of the 57 participants who initially agreed to participate in this study, two declined to participate after randomization, and eight dropped out due to health (*n* = 2), time (*n* = 1), or unknown reasons (*n* = 5). One participant of the HRV-BF group had to be excluded because of a lack of self-guided training. These individuals were predominantly female in contrast to those retained in the analysis (55% versus 15%, *p* < 0.01) but did not differ in any other variable at pre-intervention (all *p* > 0.20). In the GEE analysis of the main outcome (SDNN), four observations had to be excluded due to missing values or because the quality of the data from the 24 h Holter electrocardiogram was poor.

Table 1 summarizes participant characteristics at baseline by intervention group. There were no significant between-group differences except for age (*p* = 0.03) and LF (*p* = 0.05). Patients in the HRV-BF group were younger and had higher low-frequency power at baseline.

**Table 1:** Patient characteristics at baseline by intervention group

	HRV-biofeedback	Usual care	*p*-value^a^
	*n* = 23	*n* = 23	
Age (years)	57.4 ± 8.8	63.6 ± 9.9	0.029
Male gender	21 (91.3)	18 (78.3)	0.414
Years since last MI	5 (2;8)	3 (2;5)	0.066
Long-time measures (24 h)			
Systolic blood pressure (mm Hg)	123.9 ± 10.9	130.1 ± 12.2	0.075
SDNN (ms)	129.0 ± 47.7	130.0 ± 41.2	0.944
SDNN (ms), log	4.8 ± 0.4	4.8 ± 0.3	0.761
Short-time measures (5 min)			
Breathing rate (1/min)	12.6 ± 5.3	13.6 ± 4.2	0.478
Heart rate (bpm)	67.2 ± 12.4	64.6 ± 7.1	0.396
Heart rate (bpm), log	4.2 ± 0.2	4.2 ± 0.1	0.515
SDNN (ms)	31.7 (24;41)	24.6 (21;33)	0.118
SDNN (ms), log	3.4 ± 0.5	3.3 ± 0.3	0.177
HF	56.5 (21;120)	91.0 (49;136)	0.170
HF, log	4.0 ± 1.2	4.6 ± 1.5	0.162
LF	289.6 (94;1103)	122.7 (52;224)	0.048
LF, log	5.6 ± 1.5	4.8 ± 1.4	0.091
Psychological measures			
WHO-5 Well-being	17 (9;19)	18 (14;20)	0.223
WHO-5, squared	241.0 ± 162.5	294 ± 138.2	0.244
ASKU Self-Efficacy	4.1 ± 0.5	4.2 ± 0.4	0.225
SSCS-TICS Chronic Stress	17.7 ± 10.4	16.7 ± 9.2	0.734

Data are absolute numbers and percentages, mean values ± standard deviation, medians, and quartiles

*MI* myocardial infarction

^a^*p*-value based on Student’s *t*-test (with or without equal variances), Wilcoxon rank sum test, or Fisher’s exact test for small sample sizes

### Treatment Compliance

As we investigated a self-guided intervention, the number of home training sessions varied between the patients. The number of training sessions varied between three and 258 with a median number of 133 sessions. The recommended number of three sessions per day was fulfilled by only one patient, whereas 8 (35%) patients completed at least 168 sessions.

### Intervention Effects

Table 2 displays the means at baseline and at the end of the treatment. There was a small- to medium-sized effect of the intervention for at least three out of five short-time measures, one out of three psychological measures (each *p* < 0.05), but none of the long-time cardiovascular measures: As expected, the short-time SDNN, breathing rate, and heart rate as well as chronic stress improved significantly after HRV-BF (*d* < − 0.4 or > 0.04) but not in the control group. The difference between pre- and post-intervention in HF approached significance (*p* = 0.062) but did not do so for the controls.

**Table 2:** Observed means and Cohen’s *d* within-group effect sizes

	Group	*n*	Baseline			End of treatment		∆	*d*	*p*
			*M*	*SD*		*M*	*SD*			
Long-time measures										
SDNN (log)	HRV-BF	22	4.79	0.39		4.79	0.31	− 0.00	− 0.01	0.48
	WCG	20	4.81	0.31		4.79	0.31	0.02	0.09	0.70
Systolic BP (log)	HRV-BF	21	4.81	0.09		4.85	0.12	− 0.03	− 0.30	0.91
	WCG	22	4.86	0.09		4.88	0.11	− 0.02	− 0.16	0.46
Short-time measures										
SDNN (log)	HRV-BF	22	3.45	0.51		3.61	0.42	− 0.16	− 0.45	0.02
	WCG	17	3.28	0.34		3.21	0.35	0.08	0.23	0.35
HF (log)	HRV-BF	22	4.05	1.26		4.47	1.14	− 0.42	− 0.34	0.06
	WCG	18	4.75	1.48		4.34	1.40	0.41	0.36	0.14
LF (log)	HRV-BF	22	5.56	1.53		5.82	1.31	− 0.26	− 0.28	0.10
	WCG	18	4.98	1.25		4.61	1.37	0.37	0.32	0.20
Breathing rate	HRV-BF	22	12.69	5.37		10.16	4.80	2.53	0.53	0.01
	WCG	18	13.89	4.59		13.61	5.10	0.28	0.10	0.69
Heart rate (log)	HRV-BF	22	4.20	0.18		4.15	0.18	0.05	0.43	0.03
	WCG	18	4.16	0.12		4.19	0.16	− 0.03	− 0.21	0.39
Psychological measures										
WHO-5, squared	HRV-BF	21	250.10	160.65		262.38	127.40	− 12.29	− 0.11	0.32
	WCG	21	289.38	143.95		285.71	132.69	3.67	0.03	0.88
ASKU Self-Efficacy	HRV-BF	21	4.06	0.51		4.08	0.50	− 0.02	− 0.04	0.42
	WCG	19	4.18	0.37		4.09	0.48	0.09	0.22	0.35
SSCS-TICS Stress	HRV-BF	20	16.90	10.03		15.05	7.87	1.85	0.44	0.03
	WCG	21	16.90	9.30		14.86	8.78	2.05	0.42	0.07

*HRV-BF* heart rate variability biofeedback training, *WCG* waitlist control group

After controlling for age, gender, emotional distress, and time since last MI, the GEE models were significant for all outcomes (*p* < 0.024) except for the long-time measures of SDNN and systolic blood pressure as well as for heart rate during the short-time assessment of HRV (*p* > 0.064). The analysis showed no significant time effects (*p* > 0.050; see Table 3, left side).

**Table 3:** Adjusted time and group effects on outcomes and standardized outcomes

Outcome	All participants^a^									Optimal dose sample^a^					
	Time			Group			Group × time			Time		Group		Group × time	
	*B*	*β*	*p*	*B*	*β*	*p*	*B*	*β*	*p*	*β*	*p*	*β*	*p*	*β*	*p*
Long-time measures															
SDNN (log)	− 0.02	− 0.07	0.62	− 0.05	− 0.15	0.68	0.02	0.07	0.70	− 0.07	0.61	− 0.44	0.39	0.26	0.22
Systolic BP (log)	0.02	0.17	0.46	− 0.06	− 0.60	0.05	0.02	0.17	0.61	0.17	0.46	− 0.28	0.47	0.47	0.40
Short-time measures															
SDNN (log)	− 0.03	− 0.06	0.75	0.01	0.02	0.94	0.19	0.43	0.09	− 0.05	0.77	− 0.47	0.25	0.65	0.01
Power HF (log)	− 0.35	− 0.27	0.17	− 1.07	− 0.82	0.01	0.77	0.59	0.04	− 0.25	0.20	− 1.00	< 0.01	0.30	0.45
Power LF (log)	− 0.31	− 0.21	0.25	0.12	0.08	0.78	0.57	0.39	0.08	− 0.21	0.26	− 0.05	0.90	0.39	0.22
Breathing rate	− 0.24	− 0.05	0.72	− 0.77	− 0.16	0.57	− 2.29	− 0.48	0.06	− 0.04	0.77	− 0.53	0.21	− 0.62	0.15
Heart rate (log)	0.03	0.20	0.37	0.03	0.16	0.62	− 0.08	− 0.51	0.06	0.20	0.37	0.36	0.48	− 0.50	0.07
Psychological measures															
WHO-5, squared	− 4.36	− 0.03	0.85	− 8.19	− 0.06	0.80	17.33	0.12	0.61	− 0.03	0.86	0.01	0.97	0.24	0.30
ASKU	− 0.11	− 0.23	0.22	− 0.12	− 0.26	0.35	0.12	0.25	0.31	− 0.23	0.23	− 0.65	< 0.01	0.05	0.85
SSCS-TICS	− 2.04	− 0.23	0.05	− 1.62	− 0.18	0.47	0.13	0.01	0.93	− 0.23	0.05	0.04	0.90	− 0.15	0.40

^a^GEE models corrected for age, gender, emotional distress, and time since last MI

With respect to the differences in the outcomes between the groups over time, there was a positive group × time interaction effect (*β* = 0.59, *p* =  0.035) that qualified a negative group effect (*β* = − 0.82, *p* = 0.010) in HF. But there were only increasing trends in short-time SDNN and LF as well as decreasing trends in breathing and heart rate (see Table 3). The Bonferroni-adjusted post hoc analysis showed a significantly lower HF (*p* = 0.019) in the HRV-BF group at the beginning of the intervention as compared with the control group. But HF tended to improve in the HRV-BF group, whereas it tended to decrease in the control group. Even though these changes were not significant (*p* > 0.211), they resulted in similar HF values for the two groups at the post-intervention (*p* > 0.831).

Additional analyses with respect to compliance with the prescribed number of HRV-BF practice sessions were performed. For this purpose, GEE analyses were repeated with the waitlist control group and a selection of participants who trained on average at least two times a day (168 sessions in 12 weeks). The results of these analyses are also shown in Table 3. As with the complete data set, the Bonferroni post hoc comparisons (see Supplementary Table S3) showed a significantly lower HF (*p* = 0.003) in the HRV-BF group at baseline: trends in the opposite directions from baseline to post-intervention and no differences in either group after the intervention (*p* > 0.078). Analyses of short-time SDNN showed a significant group x time effect (*β* = 0.65, *p* = 0.012). Post hoc comparisons (see Supplementary Figure S2) displayed a highly significant increase in SDNN from pre- to post-training in the HRV-BF group (*p* = 0.002), whereas no significant change in SDNN was found in the control group (*p* > 0.999). The optimal dose analysis performed on self-efficacy yielded a negative (*β* = − 0.65, *p* = 0.004) group effect. In the post hoc comparisons, the HRV-BF group showed significantly lower self-efficacy (*p* = 0.008) at baseline, but even though there were no significant time effects (*p* > 0.450), self-efficacy no longer differed between the two groups after the intervention (*p* = 0.127).

## Discussion

In contrast to previous research, this study focused on post-MI outpatients, whereas previous studies have typically used heterogenous inpatient collectives (e.g., coronary artery disease). Thus, the present study provides better insight into opportunities for prevention after hospitalization. Furthermore, this study used mobile HRV-BF devices, whereas most previous research has used stationary devices. The use of mobile tools is important for self-guided training as part of patients’ everyday lives. Of course, a potential drawback of this flexibility is less availability of technical support and a stronger reliance on self-regulation and patient motivation.

As MI is a common disease among the elderly, our study consisted primarily of patients older than 55 years of age (32 of 46 patients). All patients but one were able and willing to conduct self-guided HRV-BF training with an average of 1.6 sessions per day. Thus, our study demonstrated the practical feasibility of mobile HRV-BF training in post-MI patients—and there was no evidence that patients might suffer any harm from conducting HRV-BF.

The results of this study confirm the beneficial effects of HRV-BF on short-time HRV indices and psychological health aspects but fail to demonstrate effects on long-time HRV indices, especially long-time SDNN as an independent cardiac risk factor.

The lack of effects on long-term cardiac measures may be due in part to our study design. First, both groups received modern pharmacotherapy, including beta blockers, ACE inhibitors, and statins, if indicated. Cardiovascular drugs can modify HRV, which renders it more difficult to detect incremental effects of HRV-BF. In order to stratify for medication, a much larger sample size would be required. Second, the usual recording of long-term HRV by wearable sensors is affected by individual daytime activities [34] and posture. In contrast to the standardized assessment of short-time HRV, the effects of HRV-BF on long-time SDNN and systolic blood pressure may be obscured by these influences, particularly because we could not control for daytime physical activity. Third, these effects on 24 h measures of HRV may take longer to evolve. Three months might not be sufficient for deep breathing or improved situation-specific HRV during biofeedback training to induce changes in habitual patterns [35] or to generalize to 24 h measures of HRV. On the other hand, the special interest in long-time SDNN is based on its usefulness in assessing cardiovascular risk. However, recent research has shown that the increase in short-time HRV is equally associated with an improved cardiovascular prognosis [23].

The effects of the intervention were clearly shown in the comparisons of the means before and after the training in short-time SDNN, breathing rate, heart rate, and chronic stress, whereas the effect on HF approached significance. In the GEE analysis, which takes the dependency of repeated observations on the same individual into account and which applied an adjustment for age, gender, time since last MI, and emotional distress, we also found evidence for some effects on short-time HRV: In contrast to the waitlist control group, patients with HRV-BF training were able to compensate for lower base levels of HF, and with at least two self-guided training sessions a day, their short-time SDNN also improved significantly.

These findings are in line with preliminary effects in stationary HRV-BF in MI patients [36] and well-documented effects in other cardiac patients [13, 22, 23]. Thus, given that there are still uncertainties regarding the outcomes of different psychological interventions for cardiovascular disease prevention [6], our study on HRV-BF adds to the understanding of the usefulness and benefits of specific psychophysiological techniques for post-MI patients. Even when controlling for emotional distress, we were able to show beneficial effects on HRV outcomes. Also, considering the low *p* values for the effect of the interaction between group allocation and time point on breathing and heart rate in the full sample (see Table 3, center columns), a consistent trend in the effects of HRV-BF on short-term measures of HRV can be assumed. There were only trends with respect to global measures of well-being such as the WHO-5, but as all participants received standard medical care when needed, the observed effects can be considered additional to the outcomes associated with modern pharmacotherapy.

Regarding self-efficacy, the results suggest that regular HRV-BF training can increase patients’ beliefs in their capabilities across a range of demanding situations. This is in line with the assumption that cognitive-attributional changes are triggered by biofeedback training (see Introduction) and extends findings on its effectiveness in improving self-efficacy in patients [37].

In patients with cardiovascular disease, low levels of generic self-efficacy are typical, and as this condition is associated with anxiety and depression, improving patients’ self-efficacy is important for improving their quality of life [38]. As we controlled for emotional distress, the effect of HRV-BF on generic self-efficacy occurred over and above improvements in depressive symptoms. Meta-analytical results have shown that targeting self-efficacy is effective in promoting health behavior [39], which suggests that this effect of HRV-BF has positive spill-over effects on other aspects of behavioral health (e.g., adherence to medication or implementation and maintenance of lifestyle changes in order to reduce individual risk factors). Given that measures of general self-efficacy beliefs usually show less predictive power than domain-specific measures [40], future studies that apply context-specific measures of self-efficacy for specific health behaviors, such as adherence to medication or coping with stress, would be desirable to further clarify the impact of HRV-BF.

### Limitations

Several limitations of the present study should be considered. First, we did not obtain the intended number of patients. Moreover, the optimal dose analyses were carried out in an even smaller subsample, and therefore, there was not enough power to detect medium-sized effects. An alternative in future research may be the use of a cross-over design. Second, observational data always bear the risk of residual confounding, and the present findings should be interpreted with caution: Clinical comorbidities and medical histories that were not captured in our study could have impacted baseline clinical differences between the HRV-BF and the waitlist control group. Another possible confound is self-selection into voluntary treatments. For example, socio-economic status, educational level, age-related cognitive impairment, comorbidities, or mobility could influence the interest and willingness to participate in the present study. However, by addressing potential candidates from an unselected outpatient registry in combination with an open call, we tried to reduce the possibilities for such selection biases. Third, we cannot totally rule out experimenter or subject artifacts, for example, in accordance with the Hawthorne effect or a frustration effect due to the waiting period in the control group. To minimize a possible experimenter artifact, the assessments of HRV and questionnaire data were not conducted by the HRV-BF trainer. It was nevertheless possible that the experimenters unconsciously influenced the responses of the participants. A double-blind design, various trainers, and additional follow-up assessments could add to the conclusiveness of the present evidence. Finally, a more profound examination of the role of breathing frequency was beyond the scope of this study. Whereas we followed recommendations to adjust the individual breathing frequency prior to HRV-BF [10] and derived resting breathing frequency from the ECG at pre- and post-intervention, we did not monitor the overall pattern of respiration (e.g., inspiration/exspiration ratio, see [10]).

Further research is needed to resolve ambiguities regarding the number and size of effects and to increase insights, for example, regarding the stability of effects after HRV-BF training ended or characteristics of the optimal target group for this type of treatment.

## Conclusions

Several large studies on secondary prevention in coronary patients have found that an optimized cardio-protective drug therapy alone does not lead to satisfactory achievements regarding risk-factor reduction (e.g. EUROASPIRE V [7]). Thus, approaches that supplement these treatments are warranted.

Mobile HRV-BF is one potential supplement that provides a safe and accessible way to improve aspects of physiological and psychological health after MI without any side-effects: The contribution of this self-guided intervention is an increase in HRV and a reduction in cardiovascular risk. Moreover, incremental benefits in self-efficacy can be regarded as a protective factor that supports patients’ abilities to cope with stressful events or health issues in the future.

Further research is needed to confirm these conclusions and to extend insights, for example, into possible mediating effects, the stability of effects, and the characteristics of the best target group for HRV-BF.

## Supplementary Information

Below is the link to the electronic supplementary material.

Supplementary file1 (DOCX 194 KB)
