# Supplementary material for: Mobile Heart Rate Variability Biofeedback as a Complementary Intervention After Myocardial Infarction: a Randomized Controlled Study
Source: Int J Behav Med. 2021 May 18;29(2):230–9. doi: 10.1007/s12529-021-10000-6 (PMC9001243; doi:10.1007/s12529-021-10000-6)
Supplement: Supplementary file 1 — Supplementary file1 (DOCX 194 KB) [file 12529_2021_10000_MOESM1_ESM.docx]

**Electronic Supplementary Material 1**


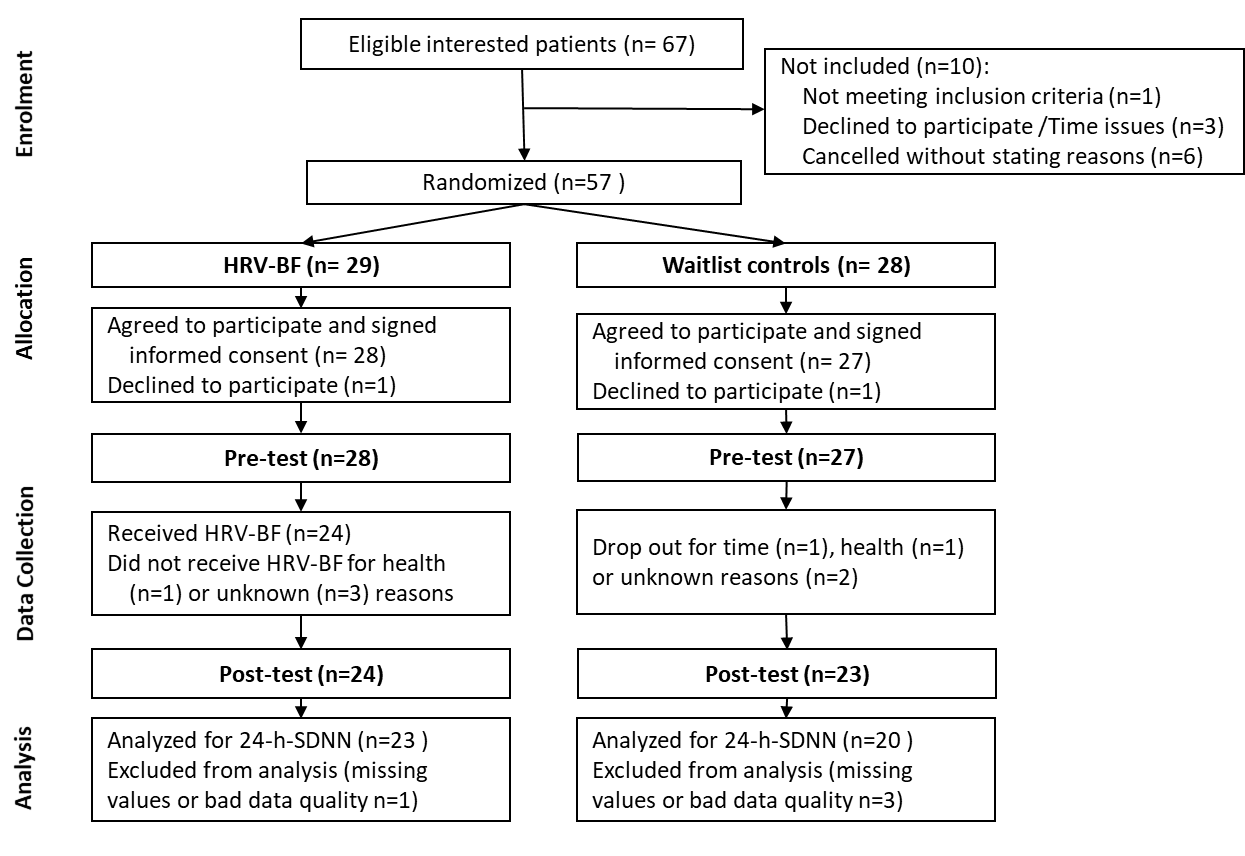


**Supplementary Figure S1.** Patient flowchart. HRV-BF: Heart rate variability biofeedback training; SDNN: standard deviation of the normal-to-normal intervals.

**Electronic Supplementary Material 2**

**Supplementary Table S2.** Average marginal effects of HRV-BF (partial group effects).

| Outcome | All participants ^a^ | | | |  | Optimal dose sample ^a^ | | |
| --- | --- | --- | --- | --- | --- | --- | --- | --- |
|  | *dy/dx* |  | | *p* |  | *dy/dx* |  | *p* |
| **Long Time Measures** | | |  | |  |  |  |  |
| SDNN (log) | -0.037 |  | | 0.722 |  | -0.100 |  | 0.508 |
| Systolic BP (log) | -0.054 |  | | 0.087 |  | -0.004 |  | 0.908 |
| **Short Time Measures** | | |  | |  |  |  |  |
| SDNN (log) | 0.102 |  | | 0.387 |  | -0.069 |  | 0.690 |
| Power HF (log) | -0.702 |  | | 0.044 |  | -1.120 |  | 0.001 |
| Power LF (log) | 0.395 |  | | 0.297 |  | 0.197 |  | 0.694 |
| Breathing rate | -1.880 |  | | 0.113 |  | -3.943 |  | 0.004 |
| Heart rate (log) | -0.014 |  | | 0.787 |  | 0.019 |  | 0.817 |
| **Psychological Measures** | | |  | |  |  |  |  |
| WHO-5, squared | 0.177 |  | | 0.995 |  | 17.963 |  | 0.693 |
| ASKU | -0.062 |  | | 0.621 |  | -0.291 |  | 0.011 |
| SSCS-TICS | -1.562 |  | | 0.433 |  | -0.239 |  | 0.933 |

^a^ GEE model corrected for age, gender, emotional distress, and time since last MI.

**Electronic Supplementary Material 3**

**Supplementary Table S3.** Contrasts in which the p-values were adjusted for multiple comparisons using the Bonferroni correction in an optimal dose sample of 31 patients (62 observations).

|  |  |  |  |  |  |  |  | Bonferroni-corrected | |
| --- | --- | --- | --- | --- | --- | --- | --- | --- | --- |
| Outcome |  | df | chi2 | p | p _corr_ | Contrast | SE | [95% Conf. Interval] | |
| Power HF | EG@time |  |  |  |  |  |  |  |  |
|  | (1 vs 0) 0 | 1 | 10.44 | 0.001 | 0.003 | -0.998 | 0.309 | -1.69 | -0.306 |
|  | (1 vs 0) 1 | 1 | 4.26 | 0.039 | 0.078 | -0.698 | 0.338 | -1.456 | 0.060 |
|  | Joint | 2 | 12.22 | 0.002 |  |  |  |  |  |
| st SDNN | EG@time |  |  |  |  |  |  |  |  |
|  | (1 vs 0) 0 | 1 | 0.08 | 0.771 | 1.000 | -0.054 | 0.185 | -0.470 | 0.362 |
|  | (1 vs 0) 1 | 1 | 10.98 | 0.001 | 0.002 | 0.593 | 0.179 | 0.192 | 0.995 |
|  | Joint | 2 | 11.07 | 0.004 |  |  |  |  |  |
| Self-Efficacy | EG@time |  |  |  |  |  |  |  |  |
|  | (1 vs 0) 0 | 1 | 8.29 | 0.004 | 0.008 | -0.650 | 0.226 | -1.155 | -0.144 |
|  | (1 vs 0) 1 | 1 | 3.44 | 0.064 | 0.127 | -0.600 | 0.324 | -1.325 | 0.125 |
|  | Joint | 2 | 8.30 | 0.016 |  |  |  |  |  |


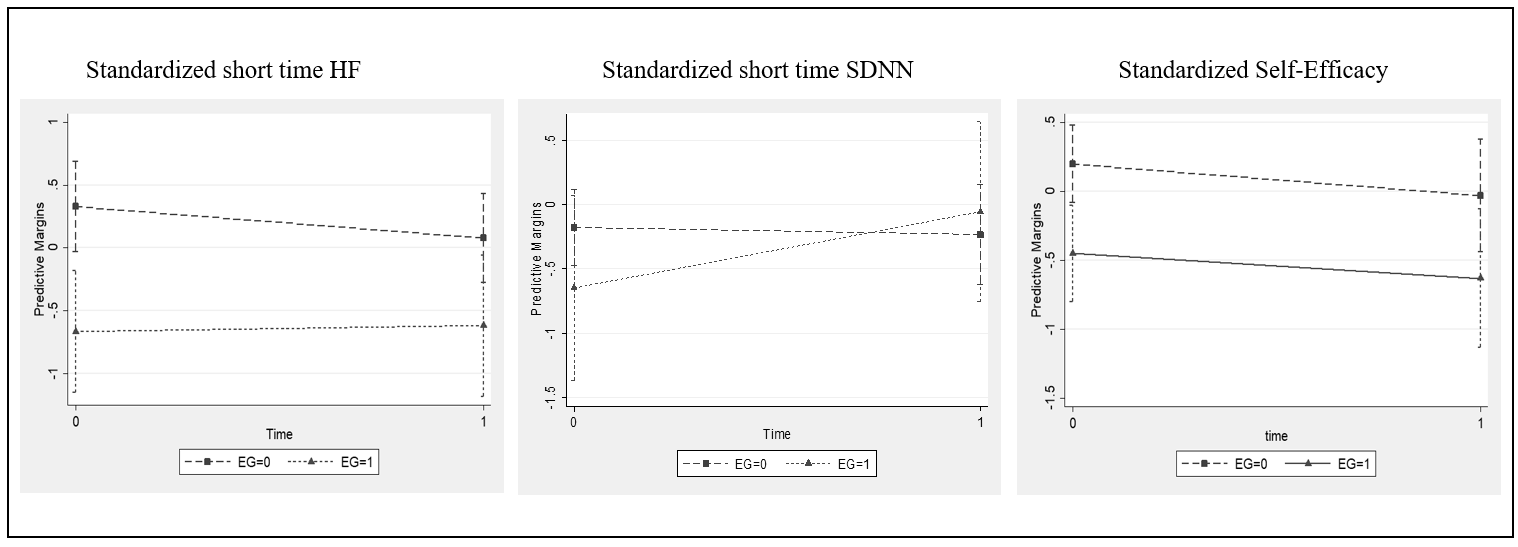


**Supplementary Figure S2.** Predictive margins for standardized outcomes with significant group or group x time effects in an optimal dose sample (EG=0: usual care; EG=1: intervention group; HF: high frequency SDNN: standard deviation of the normal-to-normal intervals).
